# Gastric cancer-associated enhancement of von Willebrand factor is regulated by vascular endothelial growth factor and related to disease severity

**DOI:** 10.1186/s12885-015-1083-6

**Published:** 2015-02-21

**Authors:** Xia Yang, Hai-jian Sun, Zhi-rong Li, Hao Zhang, Wei-jun Yang, Bing Ni, Yu-zhang Wu

**Affiliations:** 1 Institute of Immunology, Third Military Medical University, 30 Gaotanyan Street, Shapingba District, Chongqing, 400038 PR China; 2 Department of General Surgery, First People’s Hospital of Guiyang, Guiyang, 550002 PR China

**Keywords:** Von Willebrand factor, Gastric cancer, VEGF, Clinicopathological characteristics

## Abstract

**Background:**

von Willebrand factor (vWF) is a potent regulator of angiogenesis, tumor growth, and metastasis. Yet, the expression pattern of vWF in human gastric cancer (GC) tissues and its relation to clinicopathological features of these cases remains unknown.

**Methods:**

Tumor and 5-cm adjacent non-tumoral parenchyma specimens were collected from 99 patients with GC (early stages I/II and late stages III/IV), and normal specimens were collected from 32 healthy controls (reference group). Plasma vWF antigen (vWF:Ag) and vWF activity were assessed by ELISA. The role of vascular endothelial growth factor (VEGF) in differential vWF expression was investigated using cultured human umbilical vein endothelial cells (HUVECs). vWF and VEGF protein and mRNA expression levels were investigated by qRT-PCR, western blotting and immunohistochemistry (IHC) respectively. The correlation of IHC-detected vWF expression with patient clinicopathological characteristics was analyzed.

**Results:**

Compared to the reference group, the patients with late GC showed significantly higher levels of vWF:Ag (72% (21-115) vs. 101% (40-136)) and vWF activity (62% (20-112) vs. 117% (33-169)) (both *P* < 0.001). The GC tumor tissues also showed higher vWF mRNA and protein levels than the adjacent non-tumoral parenchyma. Patients at late GC stage had significantly higher median number of vWF-positive cells than patients at early GC stage (*P* < 0.05). VEGF induced vWF mRNA and protein expression in HUVECs in dose- and time-dependent manners. Patients with late GC stage also had significantly higher serum VEGF than patients at early GC stage (23 ± 26 vs. 10 ± 12 pg/mL, *P* < 0.01). Most of the undifferentiated GC tumor tissues at late disease stage exhibited strong VEGF and VEGFR2 protein staining, which co-localized with the vWF protein staining pattern.

**Conclusions:**

GC-related plasma vWF:Ag and vWF activity levels become substantially elevated in the late stage of disease. The higher mRNA and protein expression of vWF in GC tumor stroma may be regulated by the VEGF-VEGFR2 signaling pathway *in vitro* and may contribute to GC progression *in vivo*.

## Background

Gastric cancer (GC) is the second leading cause of cancer death worldwide, and the annual rate of new cases is increasing by about 1 million [1]. Over half of the reported new GC cases are from developing countries, with China accounting for a large portion of those [2]. As one of the most lethal malignant diseases, a strong correlation exists between GC and aberrant hemostasis. Concomitant thromboembolism conditions observed in GC patients include disseminated intravascular coagulation or acute disseminated intravascular coagulation [3], hemolytic-uremic syndrome [4], Budd-Chiari syndrome [5], portal vein thrombosis, intravascular coagulation, thrombotic microangiopathy, thrombotic thrombocytopenic purpura, immune thrombocytopenia, obliterative endarteritis, pulmonary thromboembolism, nonbacterial thrombotic endocarditis, and acquired factor deficiency [6]. Research on the GC-hemostasis association has revealed that the increased expression of tissue factor (TF) promotes the pathogenic conditions of coagulation, tumor growth, and angiogenesis [7].

von Willebrand factor (vWF), the macromolecular plasma glycoprotein named for its contribution to the hereditary bleeding disorder known as von Willebrand disease (vWD), functions as a key regulator of primary hemostasis. As such, vWF also represents a potential etiological factor throughout the myriad spectrum of vascular disorders, and has been implicated in thrombotic thrombocytopenic purpura clotting disorder, coronary heart disease [8], ischemia stroke [9], cerebral sinus and venous thrombosis [10], atrial fibrillation [11], hypertension [12], and sickle cell disease [13]. vWF is produced exclusively by endothelial cells and megakaryocytes. Following cleavage of the precursor prepro-vWF form, the mature vWF is stored in Weibel-Palade bodies until its release is stimulated by various secretagogues or pathological stimuli, including inflammatory factors. The circulating vWF exists in an ultra-large form (ULvWF) composed of several hundred vWF monomers which are more likely to bind platelets and collagen and therefore to promote clotting [14].

The integral link between tumorigenesis and angiogenesis supports a potential role for vWF in cancer. Indeed, studies of tumorigenic properties in a vWF-null mouse with lung cancer revealed a potential protective role for vWF against metastasis [15]. In a study of the human tissue microenvironment in non-small cell lung cancer demonstrated that the disintegrin and metalloproteinase 28 (ADAM28) can promote metastasis by binding to and cleaving vWF in carcinoma cells [16]. Moreover, a study of vWF expression in endothelial cells showed that short interfering RNA-mediated inhibition of vWF *in vitro* promoted angiogenesis and vascular endothelial growth factor (VEGF)-dependent proliferation and migration [17]. However, another human study of patients with colorectal cancer observed higher numbers of vWF-positive microvessels and a striking absence of macrophages in the tumor tissues, and suggested a positive association between these findings and poor clinical outcome [18]. While a subsequent study of tumor angiogenesis characterized vWF staining as an effective clinical maker of microvessel density, suggesting its clinical utility as a prognostic marker of cancer progression or patient survival [19], its roles in GC have not yet been fully characterized.

The present study was designed to assess the expression of vWF using *ex vivo* analysis of human specimens of GC and adjacent non-tumor parenchymal tissues and to investigate the potential molecular mechanism of GC-related differential expression of vWF using *in vitro* analysis of human umbilical vein endothelial cells (HUVECs) exposed to VEGF.

## Methods

### Patients and tissue specimens

All study procedures involving human patients and specimens were carried out with pre-approval by the Institutional Ethics Board of Chongqing Cancer Hospital. All study participants provided written informed consent prior to enrollment.

Ninety-nine patients with GC were recruited from the Department of Gastroenterological Surgery at Chongqing Cancer Hospital between 2008 and 2012. The study group consisted of 33 men and 66 females, with an average age of 57.1 ± 11.4 (range: 28-86 years). No patient had received neoadjuvant chemotherapy. GC specimens and biopsies of normal gastric mucosa (5 cm away from the tumor margin) were collected from all patients. The results of pathological analysis, including histological subtype and tumor-node-metastasis (TNM) stage, are shown in Table 1. Disease stage was classified as early (stages I and II) or late (stages III and IV). Blood samples were drawn from each patient, mixed with sodium citrate (0.129 mol/L) at a 9:1 volume ratio, and centrifuged (2,500 g for 15 min at 4°C); the resultant serum samples were stored at -80°C until use.

**Table 1:** **Clinical characteristics of 99 patients with gastric cancer**

Characteristics	No. (%)
**Age, years**	
Median	57.1 ± 11.4
Range	28-86
**Sex**	
Male	66 (66.7)
Female	33 (33.3)
**Tumor location**	
Lower stomach	50 (50.5)
Middle stomach	14 (14.1)
Upper stomach	22 (22.2)
Whole stomach	13 (13.1)
**Tumor (T) stage**	
T1	6 (6.0)
T2	16 (16.2)
T3	67 (67.7)
T4	10 (10.1)
**Lymphatic vessel invasion**	
With	70 (70.7)
Without	29 (29.3)
**Pathological lymph node (N) status**	
N0	25 (25.2)
N1	37 (37.4)
N2	34 (34.3)
N3	3 (3.0)
**Distant metastasis (M) status**	
M0	94 (94.9)
M1	5 (5.1)
**TNM stage**	
I	16 (16.2)
II	14 (14.1)
III	55 (55.6)
IV	14 (14.1)
**Histological type**	
Differentiated	30 (30.3)
Undifferentiated	69 (69.7)

### Assays to measure concentrations of serum inflammation cytokines

Serum from patients with GC were subjected to flow cytometric analysis to quantitatively assess the profiles of secreted inflammatory cytokines (including interleukin-8 (IL-8), interleukin-1β (IL-1β), interleukin-6 (IL-6), interleukin-10 (IL-10), tumor necrosis factor-alpha (TNF-α), and interleukin-12p70 (IL-12p70)) using a Cytometric Bead Array (CBA) Human Inflammatory Cytokines Kit (BD-Bioscience, San Diego, CA, USA) and the BD FACSAria flow cytometer equipped with FCAP Array analytical software (Becton, Dickinson and Company, Franklin Lakes, NJ, USA).

### Assays of vWF activity, vWF antigen (vWF:Ag) concentration, and serum VEGF concentration

The plasma control group consisted of 32 healthy subjects (15 females and 17 males) aged 21-63 years (average age: 42.2 ± 13.3). Plasma samples from the control group and the group of patients with GC were prepared by centrifuging anticoagulated blood (in 3.8 g/dL sodium citrate) specimens at 2,000 g for 15 min at 4°C, and stored in aliquots at -80°C until analysis. The plasma vWF activity was detected using a commercially available direct enzyme-linked immunosorbent assay (ELISA) kit (IMUBIND; American Diagnostica Inc., Stamford, CT, USA). The plasma vWF:Ag was quantified by sandwich ELISA using the rabbit anti-human vWF polyclonal antibody (Dako, Kyoto, Japan). Serum concentrations of VEGF were analyzed using a commercially available direct ELISA kit (NeoBioscience Technology Co. Ltd, Beijing, China).

### Cell culture

HUVECs were cultured at 37°C (humidified 5% CO_2_ atmosphere) in M-199 culture medium containing 10% fetal bovine serum (FBS), 50 μg/mL endothelial cell growth supplement (Sigma, St Louis, MO, USA), 90 μg/mL heparin (Gibco, Invitrogen, Carlsbad, CA, USA), 50 U/mL penicillin, and 50 U/mL streptomycin (Gibco, Invitrogen). After reaching confluence, the medium was replaced with an FBS-free medium and cells were incubated for an additional 2 h to achieve synchronization. The cells were then stimulated by exposure to recombinant human VEGF165 (Peprotech, Rocky Hill, NJ, USA) at various concentrations (10, 50 or 100 ng/mL in water) for various times (5, 20, 40, 80 or 120 min). Unstimulated synchronized HUVECs (0 ng/mL in water) served as controls.

### RNA isolation and real-time quantitative reverse transcription (qRT)-PCR

The mRNA expression of vWF was evaluated in GC tissues, normal tissues, and HUVECs using qRT-PCR. Briefly, total RNA was extracted using the Trizol Reagent (Invitrogen) and reverse transcribed (1 μg aliquot) using PrimeScript^TM^ Reverse Transcriptase Kit (Takara Bio Inc., Dalian, China). The resultant cDNA (2 μL) was applied as template for qPCR amplification with the SYBR Premix Ex*Taq* PCR Kit reagents (Takara Bio Inc., Dalian, China) and the following gene-specific primer pairs respectively (1 μL each; sense and antisense): vWF: 5'-TAAGTCTGAAGTAGAGGTGG-3' and 5'-AGAGCAGCAGGAGCACTGGT-3'; 18 s rRNA: 5'-CAGCCACCCGAGATTGAGCA-3' and 5'-TAGTAGCGACGGGCGGTGTG-3'. The reactions were performed on a Mx3000P real-time PCR system (Agilent Technologies Inc., Santa Clara, CA, USA) with the following thermal cycling parameters: one cycle of denaturation at 95°C for 5 min and 45 cycles of amplification consisting of denaturation at 95°C for 20 sec, annealing and extension at 60°C for 40 sec. Each sample was analyzed in triplicate. The relative levels of gene expression were calculated by the 2^-∆∆Ct^ method. Results are expressed as the ratio of vWF mRNA to the geometric average of 18 s rRNA.

### Western blot analysis

The protein expression of vWF and *β*-actin was evaluated in GC tissues, normal gastric tissues, and HUVECs by western blotting. Briefly, total protein was extracted by RIPA (Beyotime Biotechnology, Shanghai, China) and the concentration was determined by a BCA protein assay kit (Beyotime Biotechnology, Shanghai, China). Equal amounts of protein (20 μg) were resolved by SDS-PAGE and transferred onto PVDF membranes (Millipore, Billerica, MA, USA) [20]. After non-specific binding sites were blocked by a 2 h incubation with 5% milk at room temperature, the membranes were exposed to primary rabbit anti-vWF antibodies (1:800 dilutions; ab6994, Abcam, Cambridge, UK) at 4°C for overnight and anti-β-actin antibodies (1:2000 dilutions; NBL02, NeoBioscience) for 2 h at room temperature. Membranes were then washed with TBS with 0.1% Tween-20 and exposed to the appropriate horseradish peroxidase-conjugated secondary antibodies for 2 h at room temperature. The bands were visualized by using Digital Imaging System (Carestream Image Station 4000MM, Carestream Health, Inc) with ECL substrate (Beyotime Biotechnology, Shanghai, China).

### Immunohistochemistry (IHC)

The human tissue specimens were formalin-fixed, paraffin-embedded, and sectioned (4 μm thickness). For IHC, the sections were deparaffinized thoroughly by xylene and then rehydrated through an alcohol gradient. Antigen retrieval was carried out by immersing the samples in pre-heated (90°C) EDTA (pH 8.0) for VEGF detection or citrated buffer for all other antigens’ detection, and heated (by microwave) at 95°C for 20 min. After cooling to room temperature, the samples were thoroughly washed with PBS and exposed to 5% H_2_O_2_ in 50% methanol at room temperature for 1 h to block endogenous peroxidase activities and goat serum at 4°C for 30 min to block non-specific binding sites. Then, the samples were exposed to the primary antibodies rabbit anti-vWF (1:400; ab6994, Abcam), rabbit anti-CD31 (1:100; ab28364, Abcam), mouse anti-VEGF and anti-FVIII (1:1; Maixin-Bio, Fuzhou, China), and rabbit anti-VEGFR2 (1:2; Zhongshan Golden Bridge Biotechnology, Beijing, China) at 4°C overnight in a humidity box. After a triplicate PBS wash, the immunostaining was visualized by DAB and hematoxylin. Negative controls were generated using the same procedure but with the primary antibodies of mouse anti-IgG1 (Dako) and normal goat IgG (Santa Cruz Biotechnology Inc., Santa Cruz, CA, USA).

The mean amount of positive-staining cells in each sample was determined by averaging the numbers from five separate high-power microscopic field (HPF) regions (×200; BX51 microscope, Olympus, Tokyo, Japan).

### Statistical analysis

All statistical analyses were carried out with the SPSS v13.0 software (SPSS Inc., Chicago, IL, USA). Inter-group differences were evaluated by the Student's *t*-test, with the threshold of statistical significance represented by a *P*-value of <0.05. The correlation analysis between vWF, VEGF, VEGFR2 and clinicopathologic variables of GC was evaluated by Wilcoxon rank sum test or Kruskal-Wallis *H* test.

## Results

### GC tissues show substantially elevated levels of vWF:Ag and vWF activity in plasma

**Figure 1:**
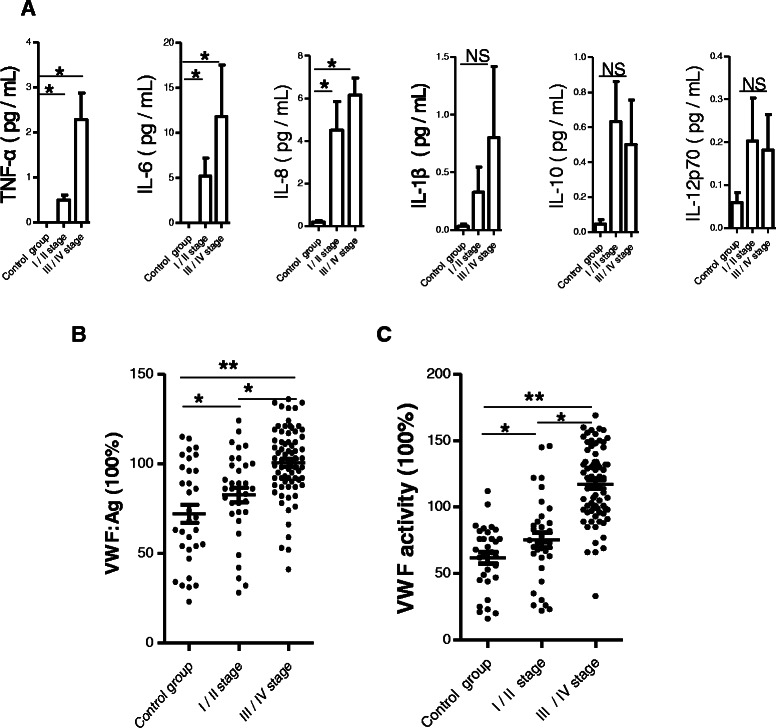
**Patients with GC have elevated levels of** IL-6, IL-8, TNF-α and **vWF in plasma. (A)** Inflammatory cytokines measured included IL-1β, IL-6, IL-8, IL-10, TNF-α and IL-12p70. **(B)** VWF:Ag and **(C)** VWF activity levels in control group and patients with early disease (I/II stages) and late disease (III/IV stage). Data are expressed as percentages of the respective vWF parameter measured in the healthy control group. Horizontal lines represent medians. *p < 0.05 and **p < 0.01 by Student's *t*-test. NS, non-significant.

Compared to the healthy controls, patients with GC showed higher levels of the secreted cytokines IL-6, IL-8 and TNF-α (all *P* < 0.05) (Figure 1A); the levels of IL-1β, IL-10 and IL-12p70 were not significantly different between the two groups. Compared to the healthy controls (median: 72% [range: 21-115]), the patients with GC showed significantly enhanced plasma vWF:Ag levels (*P* < 0.05 for all patients with GC) (Figure 1B). Moreover, the GC-related increase in plasma vWF:Ag levels was associated with disease severity, with patients with late disease stage showing higher levels than patients with early disease stage (101% [40-136] and 82% [8-118] vs. healthy controls, *P* < 0.001).

A similar trend was seen in the plasma vWF activity levels, where the levels were significantly enhanced in patients with GC (vs. healthy controls: 62% [20-112], *P* < 0.01) and followed the disease severity (late disease stage: 117% [33-169] and early disease stage: 75% [22-145] vs. healthy controls, *P* < 0.001) (Figure 1C).

### Gastrointestinal stromal tumors show increased expression levels of vWF

**Figure 2:**
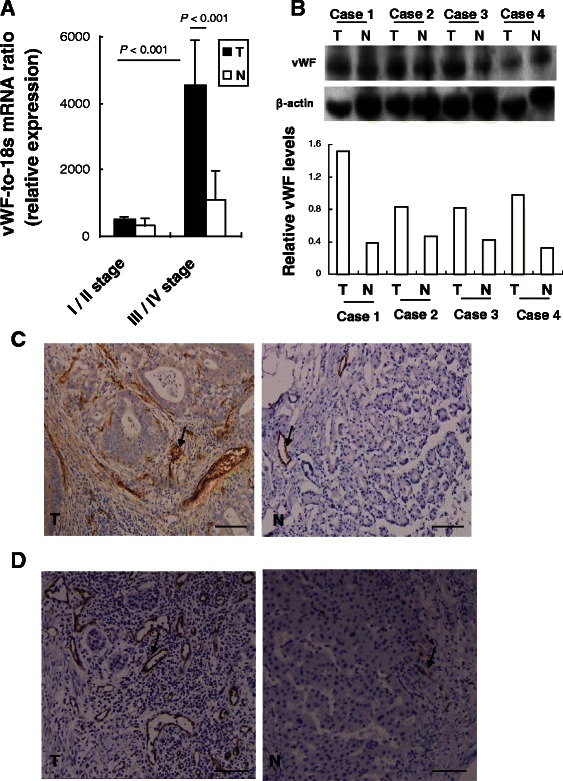
**GC tumor specimens have elevated levels of vWF expression. (A)** qRT-PCR detected levels of vWF mRNA. Data are presented as relative Ct values from the GC tumor samples and patient-matched adjacent normal tissue samples (*n* = 32). **(B)** Western blot detected levels of vWF protein in GC tumor samples and patient-matched adjacent normal tissue samples (upper panel). The relative expression level of vWF protein is shown, normalized to the β-actin loading control (lower panel). IHC detected levels of **(C)** vWF and **(D)** FVIII protein (brown: positive cells) in a representative GC tumor sample and the patient-matched adjacent normal tissue sample. Magnification: ×200. Bar =100 μm. T, tumor sample; N, normal sample.

In the patients with GC, the level of vWF expression was significantly higher in the tumor tissues than in the adjacent normal tissues, at both the mRNA (Figure 2A) and protein (Figure 2B) levels. In addition, IHC detected remarkably higher levels of vWF protein expression concentrated in the tumor stroma region (Figure 2C). Interestingly, the expression of FVIII protein was expressed in microvascular of tumor stroma region, and consistent with the expression of vWF protein (Figure 2D).

### Patients with GC have elevated serum levels of VEGF and VEGF treatment induces vWF mRNA and protein expression in the HUVEC endothelial cell line

**Figure 3:**
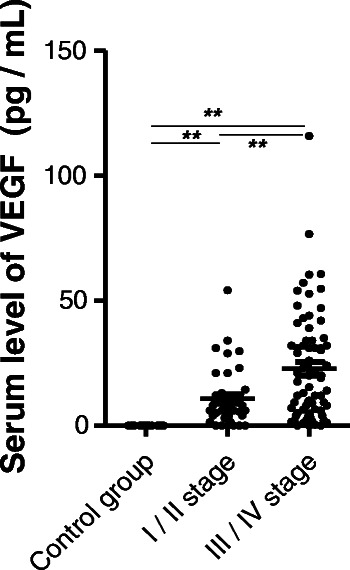
**Patients with GC had elevated levels of VEGF in plasma.** Serum levels of VEGF were measured in patients with GC with early disease (I/II stages) and late disease (III/IV stages) by ELISA. **p < 0.01 by Student's *t*-test.

Enhanced serum VEGF was detected in the patients with GC upon hospital admission; in comparison, the healthy controls had undetectable levels of VEGF in serum (data not shown). When the GC-related enhanced levels of serum VEGF were evaluated in accordance of disease state, it was found that patients with late disease had significantly higher levels than those with early disease (23 ± 26 pg/mL vs. 10 ± 12 pg/mL, *P* < 0.01) (Figure 3).

**Figure 4:**
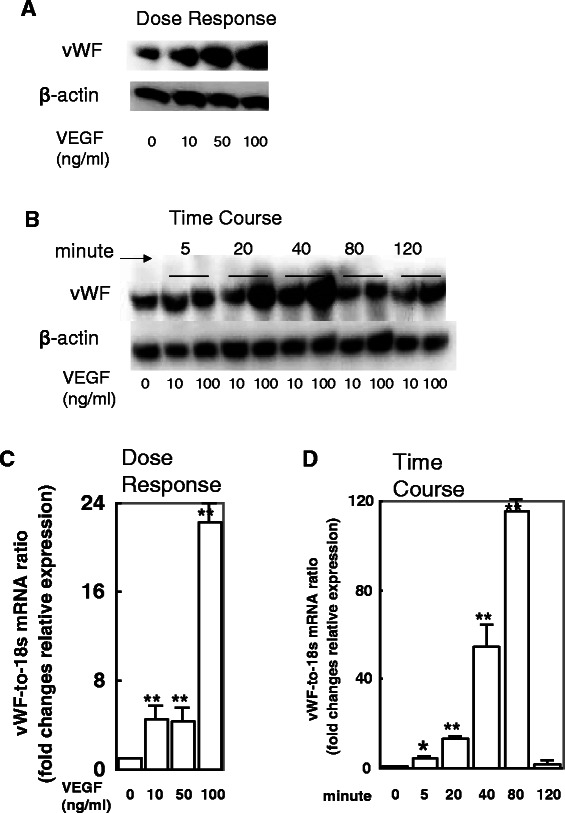
**VEGF treatment induces vWF expression in and secretion from HUVEC cell lines in a dose- and time-dependent manner.** HUVECs were exposed to 0, 10, 50 or 100 ng/mL of VEGF for 1 h and examined by western blot **(A)** and qRT-PCR **(C)**. The concentration of 100 ng/mL induced the highest level of vWF protein and mRNA expression. HUVECs were exposed to the various doses of VEGF for the indicated times and examined by western blot **(B)** and qRT-PCR **(D)**. The exposure time of 40 minutes stimulated the highest level of vWF protein expression but 80 minutes stimulated the highest level of vWF mRNA expression. *p < 0.05 and **p < 0.01 by Student's *t*-test.

To investigate the potential impact of up-regulated VEGF on vWF expression, HUVECs were treated with different doses and times of VEGF and the changes in vWF gene and protein expression were examined. The highest level of vWF protein expression occurred upon exposure to the highest concentration of VEGF (100 ng/mL), showing a dose-dependent response trend for VEGF effects on vWF protein (Figure 4A). Similarly, the highest level of vWF protein occurred after the 40 minute exposure time, suggesting a time-dependent response trend for VEGF effects on vWF protein (Figure 4B). Similar dose- and time-dependent responses to VEGF were observed for vWF at the mRNA level (Figure 4C, 4D).

### Intratumoral distribution of vWF, VEGF and VEGFR2 expression and the relationship with GC clinicopathological features

**Figure 5:**
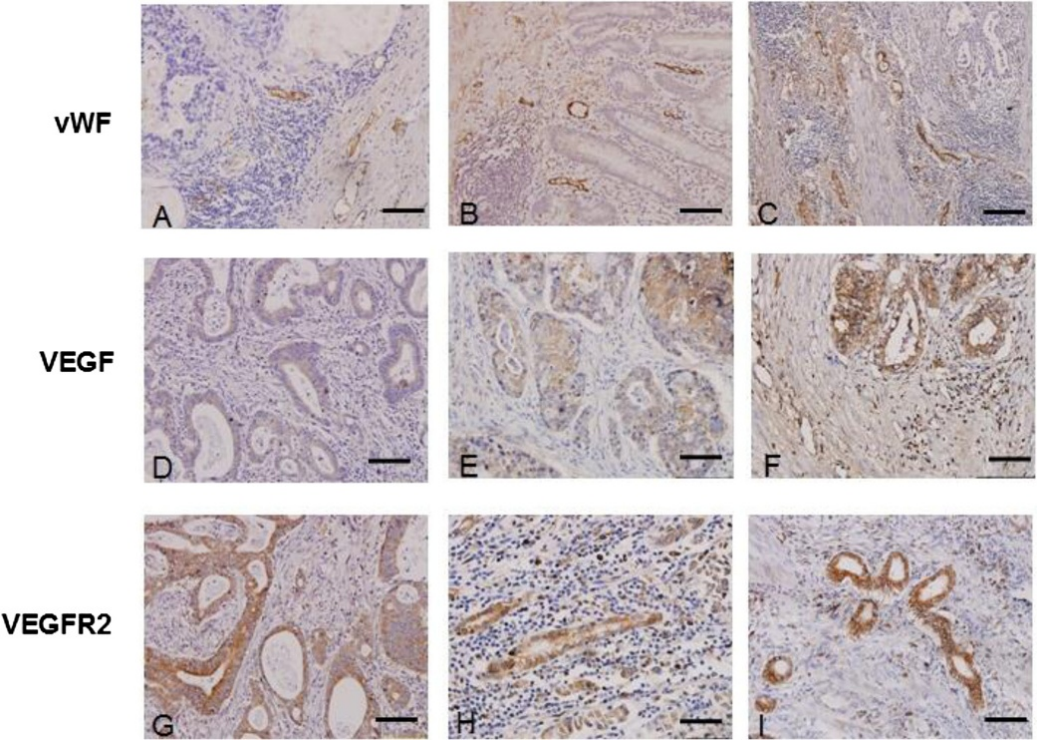
**IHC staining patterns of vWF, VEGF, and VEGFR2 in GC tissues.** The following representative tumors are presented: **(A, D, G)** well-differentiated with TNM stage II; (B, E, H) undifferentiated tumor with TNM stage II; **(C, F, I)** undifferentiated tumor with TNM stage IIIb, IIIa and IV respectively. vWF staining is shown in **(A-C)**. VEGF staining is shown in **(D-F)**. VEGFR2 staining is shown in **(G-I)**. Positive cells are stained brown. Magnification: ×200. Bar = 100 μm.

The IHC staining patterns of vWF, VEGF, and VEGFR2 and corresponding GC clinicopathological features are presented in Table 2. vWF immunostaining was highest around the tumor nests, where microvessel density (MVD) was highest as well. Compared to patient-matched adjacent non-tumor tissues (Figure 2C), the tumor tissues from patients with early stage disease showed slightly increased MVD (Figure 5A, B) while those from patients with late stage disease showed markedly increased MVD (Figure 5C). The number of cells showing vWF-positive staining was significantly higher in the patients with late disease stage disease than in those with early stage disease (*P* < 0.05). No relationship was found between the level of vWF-positive staining and patient sex, age, presence of lymph node metastasis or extent of tumor differentiation (all *P* > 0.05).

**Table 2:** **Immunohistochemical staining of the vWF, VEGF, or VEGFR2 and clinicopathological characteristics in patients with gastric cancer**

Variables	vWF-positive			VEGF-positive			VEGFR2-positive		
	≤125.75	>125.75	*p*-value	≤68.12	>68.12	*p*-value	≤114.56	>114.56	*p*-value
**Sex**									
Female	15	18		17	16		14	19	
Male	31	35	0.619	32	34	0.851	30	36	0.307
**Age, years**									
≥57	23	30		25	28		29	24	
<57	21	25	0.256	24	22	0.736	22	24	0.674
**Lymph node metastasis**									
No	10	15		14	11		12	13	
Yes	32	42	0.059	35	39	0.692	40	34	0.716
**TNM stage**									
I + II	20	10		16	14		15	15	
III + IV	20	49	0.019*	20	49	0.016*	19	50	0.013*
**Histological type**									
Differentiated	13	17		18	12		16	14	
Undifferentiated	30	39	0.173	25	44	0.043*	23	46	0.023*

*p < 0.05; statistical significant values indicated by bold font.

VEGF and VEGFR2 cytoplasmic immunostaining was detected in all cancer cells in tumor tissues (Figure 5D-I). The late stage disease and undifferentiated tumor tissues from patients with late disease stage exhibited higher levels of VEGF and VEGFR2 (Figure 5F and I) than those from patients with early disease stage (Figure 5E and H). In addition, the quantity of cells showing VEGF-positive and VEGFR2-positive staining was significantly higher in those patients (*P* < 0.05) (Table 2). A higher number of vWF-positive cells was associated with a higher number of VEGF-positive and VEGFR2-positive cells in the patients with late disease stage.

## Discussion

Extensive research efforts have been put forth to help elucidate the dynamic and critical roles of vWF in hemostatic and thrombotic processes; however, much less research into its roles in GC pathogenesis has been conducted and much fewer data have been reported. The study described herein represents the first clinical report of the GC-related vWF expression pattern and its clinicopathological significance for humans. The data from this study not only provide novel insights into the likely role of vWF in GC pathogenesis, but also highlight the potential clinical significance of serum vWF and tumor-related mRNA and protein expression as markers of disease stage and prognosis.

Specifically, patients with GC were shown to have enhanced levels of vWF:Ag and vWF activity in plasma and a strong correlation was observed between these two variables and disease severity. These findings are similar to previous data from patients with colorectal cancer, who showed elevated plasma vWF that correlated with metastatic potential [21]. Interestingly, a previous study of lung cancer showed that ADAM28 can downregulate vWF and cleave proapoptotic VWF in carcinoma cells, thereby increasing lung metastasis [16]. Data from mouse models (vWF-null) and cultured endothelial cells have supported the potential of a protective role for vWF against metastasis [15,17].

Considering that vWF may act as a key factor in resistance to metastasis and also as an inhibitor of angiogenesis, vWF may be a useful progrognostic marker; however, data from other studies have indicated that it may not be a general marker for all cancer types. Studies of non-small cell lung cancer patients and breast cancer patients found no substantial alterations in vWF:Ag levels compared to reference controls [22,23], and a clinical trial of human patients with colorectal cancer found significantly elevated levels of plasma vWF but was unable to clearly define the related role in cancer progression [24]. It is possible that perturbed plasma vWF:Ag levels may be more indicative of organ-specific processes, general risk factors, or pathogenic states associated with comorbidities. Indeed, elevated plasma vWF:Ag levels have been reported in cases of acute liver injury/failure, alcoholic hepatitis, liver cirrhosis, and sickle cell disease [13,25-27]. Conway *et al.* also showed that elevated vWF:Ag levels were independently associated with advanced age, prior cerebral ischemia, recent heart failure, diabetes, and non-valvular atrial fibrillation [28,29]. Ongoing investigations in our laboratory have indicated that patients with liver cirrhosis show even higher levels of elevated vWF:Ag and vWF activity than the patients with GC reported herein (data not shown). Thus, pathogenesis-related elevations in plasma vWF may be related to endothelial dysfunction. Since the collective data have yet to provide a precise profile of elevated serum vWF, it cannot be recommended as a clinical marker of GC.

Similar to the elevated vWF protein expression profile observed in human GC tissues, the current study also observed elevated vWF mRNA expression. Furthermore, the elevated expression was most robust in the tumors’ stromal regions and in late disease stage. In a previous study of colon carcinoma specimens, almost all (5/6) were found to possess higher vWF mRNA levels than their patient-matched normal tissues [30]. vWF IHC staining represents an effective maker of MVD, and as such has been suggested that as a useful prognostic marker for colorectal, ovarian and prostate cancers’ progression and/or patient survival [18,31,32]. In particular, the vWF IHC staining in ovarian solid carcinoma was shown to be associated with poor survival [33]. In another study based on the HUVEC cell line, it was shown that the VEGF-VEGFR2 pathway was able to induce the release of full-length vWF, and that this process involved cAMP/protein kinase A (PKA) signaling [34]. While the results from the present study are in agreement with these previous findings the precise functional mechanism of vWF in tumorigenesis and tumor progression remain far from being completely understood.

Our *in vitro*-based data revealed a possible functional network involving VEGF signaling and vWF expression in human GC, which our *ex vivo* experiments indicated was also related to severity of disease state. Moreover, the IHC-observed co-localization of VEGF and VEGFR2 molecules with GC-elevated vWF proteins further supported the theory that these factors may represent a mechanism of GC pathogenesis (and possible target of future molecular therapies). A recent study indicated that vWF may play a protective role by promoting resistance to tumor cell metastasis and dissemination *in vivo* [35], lending further support to the promising potential of vWF manipulation while highlighting the fact that our understanding of the molecular basis for achieving such a therapeutic effect remains largely incomplete. The data in the current study serves to justify the continuance of such vWF-focused studies, especially in GC.

## Conclusions

In conclusion, plasma vWF:Ag and vWF activity levels are substantially elevated in patients with GC, especially in those who have reached the late stage of the disease condition. The particularly robust enhancement of vWF protein and mRNA expression in stromal regions of GC tumors, along with the physical proximity and functional relationship to the VEGF-VEGFR2 molecules and signaling pathway, suggest a potential pathogenic mechanism of GC and targets of future molecular therapies.
